# Electroanalysis of Biochemistry and Material Chemistry—2nd Edition

**DOI:** 10.3390/molecules31091474

**Published:** 2026-04-29

**Authors:** Guangjin Wang

**Affiliations:** School of Materials and Energy, Foshan University, Foshan 528051, China; wgj501@163.com

## 1. Introduction

Building on the success of the first edition of the Special Issue, entitled “Electroanalysis of Biochemistry and Material Chemistry”, and with the aim of collecting more recent research advances in biochemistry and materials chemistry, the second edition of the Special Issue has been launched in *Molecules*. Through the joint efforts of reviewers and editors, 11 papers—including eight research articles and three reviews—have been published in this Special Issue. Herein, a brief review of the latest findings of the abovementioned published papers has been summarized in the following section.

## 2. An Overview of the Published Articles

Owing to their outstanding physicochemical properties, including high hardness, high melting point and excellent chemical and electrochemical corrosion resistance, transition metal carbides have been employed as reinforcing phases in coating materials to enhance the mechanical properties of metallic substrates [1]. In Contribution 1, Jiang et al. systematically reviewed the preparation methods, structural characteristics and application performance of five typical transition metal carbide-reinforced coatings, including those strengthened with WC, TiC, NbC, Ti_n+1_AlC_n_ (MAX phases) and Cr_3_C_2_-TaC. Subsequently, Jiang et al. (Contribution 2) investigated the impact of Y_2_O_3_ usage on the hardness, wear resistance and electrochemical corrosion resistance of Fe60/WC/Y_2_O_3_ coating prepared by laser cladding on 42CrMo steel substrates. It was found that the introduction of Y_2_O_3_ accelerated the decomposition of metal carbides to form gaseous products, thereby promoting the formation of a porous structure within the coating matrix. When the Y_2_O_3_ content reached 2.5 wt.%, the coating achieved a hardness of 861.97 HV, a friction coefficient of 0.675 and a wear volume of 1.8 × 10^−3^ mm^3^. This sample also exhibited excellent electrochemical corrosion resistance, with a corrosion potential of −0.704 V and a corrosion current density of 0.013 mA cm^−2^. The usage of additives, as well as their distribution in matrices, impacts material physicochemical properties [2]. Wang et al. (Contribution 3) reported the relationship between the distribution of Tb and Ga in sintered Nd-Fe-B magnet matrices and their magnetic properties. It was found that the introduction of Ga promoted the diffusion of Tb within the magnet matrices, thereby enhancing their coercivity, and the simultaneous introduction of Tb and Ga into the magnet matrices induced the formation of a (Nd,Tb)_2_Fe_14_B shell–core structure, improving the matrices’ coercivity by 53.15%.

Post-processing time is another critical factor affecting the physicochemical properties of materials [3]. Yan et al. (Contribution 4) discussed the effect of chromizing time on the composition and microstructure of chromized layers coated on GCr15 bearing steel by powder pack chromizing. It was found that extending the chromizing treatment time hardly altered the distribution profiles of Cr, Fe, and C, or the main crystalline phases (i.e., (Cr,Fe)_23_C_6_ and (Cr,Fe)_7_C_3_) of the coatings, but significantly increased their thickness. The hardness and brittleness of the coatings increased in tandem with the chromizing time, whereas the adhesion strength between the chromized coating and the GCr15 bearing steel substrate deteriorated. As compared to the bare GCr15 bearing steel substrate, the wear resistance of the chromized sample was improved by approximately five times under the optimal chromizing time due to the high hardness of the coatings and the robust metallurgical bonding between the chromized coating and the GCr15 substrate. In addition, Niu et al. (Contribution 5) applied Nb as a strengthening additive to regulate the microstructure of NiTi alloy by the formation of dual-phase networks, thereby enhancing the electrochemical performance of NiTi alloy bipolar plates for proton exchange membrane fuel cells. Electrochemical analysis revealed that the corrosion resistance of the Nb-doped NiTi alloy was significantly improved. Under typical simulated operating conditions for proton exchange membrane fuel cells, its corrosion current density and interfacial contact resistance accounted for only 74% and 71% of those of the NiTi alloy, respectively.

On the other hand, doping with heterogeneous elements is also one of the most important methods to improve the application performance of materials [4]. Wu et al. (Contribution 6) reported the physicochemical and electrochemical properties of Bi^3+^-doped Ti/Sb-SnO_2_/PbO_2_ electrode prepared by electrodeposition. It was found that Bi^3+^ doping endowed the electrode with excellent hydrophilicity (a contact angle of 21.6°), a long service life of approximately 2000 h, a high electrochemical active area corresponding to a total voltammetric charge of 21.20 C cm^−2^, and outstanding electrocatalytic activity for the oxygen evolution with an onset potential as low as 1.80 V. Its superior electrochemical performance in zinc electrowinning was attributed to its high electron transfer capability, reflected by the lowest charge transfer resistance of 7.30 Ω. In addition, first-principles calculations were employed to reveal the underlying mechanism responsible for the enhanced electrochemical performance of Bi^3+^-doped electrodes. The calculated results demonstrated Bi^3+^ doping reduced the band gap of the as-prepared electrode, thereby improving its electronical conductivity. Moreover, the formation of OOH determined the reaction rate of the oxygen evolution reaction on the surface of the electrode, with a free energy barrier of 2.23 eV. In addition, hydrophilicity is employed to evaluate the performance of electrode materials in a redox flow battery [5]. For example, Mei et al. (Contribution 7) prepared highly hydrophilic nanorod-like NiMoS-modified carbon felt composites using one-step hydrothermal method, with a contact angle of 140°. Electrochemical measurements revealed that the charge transfer resistance of the as-prepared composites was 524 mΩ. When employed as the anode in a polysulfide-ferricyanide redox flow battery, the composites delivered an energy efficiency of 70% and a voltage efficiency of 87% in the first cycle, at a current density of 40 mA cm^−2^. Under the same current density, the assembled battery maintained an energy efficiency of 40% after 2500 charge–discharge cycles, with a retained average coulombic efficiency as high as 99.9%.

Due to their large specific surface area, high porosity, well-defined crystal structure and tunable functionalized surfaces and good biological compatibility, metal–organic frames (MOFs) and their derivatives have attracted considerable attention in energy storage and electrochemical sensing applications [6]. Therefore, Suganthi et al. (Contribution 8) systematically reviewed the synthetic strategies of MOFs and MOF/MXene hybrids, as well as their supercapacitive performance and electrochemical detection performance for various hazardous substances, including heavy metal ions and picric acid. Meanwhile, based on the self-dissociation feature of metallic Ni in terephthalic acid solution, Li et al. (Contribution 9) prepared Ni-MOF microbelt arrays and applied them as the electrode materials for supercapacitors. It was found that the unique array structure endowed the as-prepared microbelt arrays with excellent electrochemical properties, achieving a capacitance of 1124 F g^−1^ at 1 A g^−1^, while still retaining 590 F g^−1^ even at a high current density up to 10 A g^−1^.

Apart from the above-mentioned contributions, Li et al. (Contribution 10) investigated the effect of the corona discharge treatment on the microstructure and chemical properties of polyethylene using various spectroscopic characterization techniques. Prolonging the increased the thickness of the corona degradation layer was found, which led to a decrease in the S parameter and an increase in the W parameter. This phenomenon was attributed to the reduction in free volume elements and the formation of oxygen-containing groups, including hydroxyl, carbonyl and ester groups. Furthermore, Ma et al. (Contribution 11) systematically reviewed the research progress of enzymatic electrochemical sensors for the detection of organophosphorus pesticides, with a focus on carbon-based, polymer-based, metal-based, metal compound-based, MOF-based and covalent organic framework-based electrochemical active materials, as well as their key performance indicators, including sensitivity, selectivity, storage stability, repeatability, reproducibility, anti-interference ability and recovery in real samples.

## 3. Conclusions

This Special Issue has collected important research findings across the following areas: (1) the influence of additive usage and distribution, postprocessing duration and second-phase formation on the physicochemical properties of alloy materials; (2) the effect of heterogeneous element doping and microstructure modulation on the electrochemical corrosion resistance of polycompounds; (3) the synthetic strategies and the supercapacitive performance of MOF-based materials; (4) the impact of corona discharge treatment on the physicochemical properties of polyethylene; and (5) the recent advances in the active materials of enzymatic electrochemical sensors for the detection of organophosphorus pesticides. The findings provide valuable references for researchers working in electrochemistry and materials chemistry. Currently, the third edition of the Special Issue entitled “Electroanalysis of Biochemistry and Material Chemistry” has been launched in Molecules. Researchers are welcome to submit their latest original work to this new Special Issue.
